# *Bacillus* sp. QSI-1 Modulate Quorum Sensing Signals Reduce *Aeromonas hydrophila* Level and Alter Gut Microbial Community Structure in Fish

**DOI:** 10.3389/fcimb.2016.00184

**Published:** 2016-12-12

**Authors:** Shuxin Zhou, An Zhang, Hongping Yin, Weihua Chu

**Affiliations:** Department of Pharmaceutical Microbiology, School of Life Science and Technology, China Pharmaceutical UniversityNanjing, China

**Keywords:** quorum quenching, N-acyl-homoserine lactones, probiotics, gut microbiota, *A. hydrophila*

## Abstract

Quorum sensing (QS) is a cell density dependent process that enables bacteria to communicate with each other based on the production, secretion and sensing of the auto-inducer molecules and then subsequently regulate virulence associated gene expression. Interrupting quorum sensing may represent a novel alternative approach to combat bacterial pathogen. Several bacteria can produce quorum quenching (QQ) enzymes. However, the role of QQ bacteria in shaping the microbiota and the level of N-acyl-homoserine lactones (AHLs, a prevalent type of QS molecules) producing bacteria remains largely unknown. The objective of this study was to examine the presence of AHLs in the fish intestine and investigate the modulation of gut microbiota and its effect on *Aeromonas hydrophila* level by a QQ enzyme producing probiotic *Bacillus* sp. QSI-1. AHLs were found in fish gut content and were confirmed in *Aeromonas* species using *Chromobacterium violaceum* CV026 and *Agrobacterium tumefaciens* AT 136 (pZLR4) as reporter strains. We demonstrated that the composition of fish gut microbiota was affected by quenching bacteria QSI-1, and the percentage of *A. hydrophila* was decreased significantly. Taken together, these results provide valuable insights into QQ enzyme producing probiotics can modulate the microbiota structure and decrease the percentage of AHL-producing pathogenic bacteria in the gut. These data strongly suggest that QQ probiotics may serve as non-antibiotic feed additive in aquaculture to control bacterial diseases.

## Introduction

The fish gastrointestinal (GI) tract is a complex ecosystem that always possessing a diverse bacterial community in a balanced relationship with each other, and some of the bacteria likely provide beneficial effects to the host (Wu et al., 2012b). It is considered that the fish gut harbors about 10^7^–10^8^ colony-forming units (CFU) g^−1^ bacteria (Pérez et al., 2010). The freshwater fish intestinal microbiota tends to be dominated by members of the genera such as *Aeromonas, Acinetobacter, Lactococcus, Flavobacterium* and *Pseudomonas*, representatives of the family *Enterobacteriaceae*, and obligate anaerobic bacteria of the genera *Clostridium, Bacteroides*, and *Fusobacterium*. Among these bacteria, their population is influenced by the metabolites and the effectors that promote species stability, adaptation, and survival in the gut (Ye et al., 2014; Kashinskaya et al., 2015). Quorum sensing (QS) perhaps plays an important role in these processes, which provide bacteria with the ability to communicate with the same or other species and change behaviors including, but not limited to, motility, biofilm formation, virulence factors secretion and extracellular digestive enzymes production, to modulate the population growth and survival in the environments. (Bassler and Losick, 2006). QS is a mechanism of cell-to-cell signaling that involves hormone-like compounds called autoinducers that the bacteria used to sense the populations and that subsequently regulate QS controlled gene expression. Acyl-homoserine lactones (AHLs) are considered to be intraspecies signaling molecules in Gram-negative bacteria (Bandara et al., 2012), although it has been shown that between bacteria using these molecules to communicate with other species (Riedel et al., 2001; Ryan and Dow, 2008; Giaouris et al., 2015).

Quorum quenching (QQ), namely the enzymatic interruption of QS, refers to the activity of autoinducers be enzymatic degradated and thereby inhibiting the gene expression controlling bacterial behaviors. Quenching QS has been recommended as a promising non-antibiotics strategy for bacterial diseases therapy (Tang and Zhang, 2014). Most pathogenic bacteria in fish, such as *Aeromonas* spp. *Edwardsiella tarda* and *Vibrio* spp. are opportunistic bacterial pathogens (Austin and Austin, 2007). The overgrowth of these bacterial pathogens in the intestine may disturb the fish core microbiome and then cause diseases (Wu et al., 2012a). AHLs are the main signal molecules produced by these fish pathogens (Swift et al., 1997; Defoirdt, 2014). Thus, QQ may be important for the host to maintain balance within gut ecosystem of micro-organisms and inhibit the virulence gene expression of pathogenic bacteria in intestinal environment.

We hypothesized that cell-cell communication through AHLs might also occur between commensal members of the gut microbiota and tried to investigate whether disruption of AHLs can shape the species composition of the community. To test our hypothesis, quorum quenching enzyme producing strain *Bacillus* sp. QSI-1 was used to evaluate its effect on the AHLs production and microbial composition of gut environments in fish. The *Bacillus* sp. QSI-1 strain is a probiotic strain isolated from the gut of a healthy fish that has been reported to have the effect of protection fish from *Aeromonas hydrophila* infection (Chu et al., 2014). Due to the complex interactions within the gut microbial community, the effects of *Bacillus* sp. QSI-1 on the QS molecules production and the composition of gut microbiota are still not known. To our knowledge this is the first study to characterize the influence of QQ enzyme producing probiotics on microbial community and the relationship with fish potential pathogen within fish. We found that supplementation of the *Bacillus* sp. QSI-1 can altere microbial community, decrease the AHLs production and reduce the level of AHL-producing bacteria –*A. hydrophila* in fish gut.

## Materials and methods

### Experimental fish and rearing

Mixed sexes healthy Goldfish (*Carassius auratus*), showing no clinical signs of disease and having a mean body length 10.4 ± 0.45 cm and weight 15.2 ± 0. 53 g were purchased from a local farm at Jiangsu province, China and maintained for 10 days in 60 L tanks containing aerated fresh water at a temperature of 28 ± 2.0°C, about 80% water in the aquariums was exchanged every week. The fish were fed with 3% basal diet per body weight, twice a day. This experiment was approved by the China Pharmaceutical University Animal Care and Use Committee.

### Basal diet preparation

The basal pelleted diet comprising 25% soybean meal, 15% fish meal, 20% rice bran, 5% corn starch, 30% rapeseed oil cake, 3.5% soybean oil and 1.5% vitamin and mineral mixture was prepared. The ingredients of the basal feed were analyzed according to the AOAC (Association of Official Analytical Chemists, 1997) method which revealed 2% moisture, 29.8% crude protein, 5.29% crude fat and 13.1% ash. For the preparation the diets with *Bacillus* sp. QSI-1, QSI-1 were grown in nutrient broth medium with aeration in a shaking incubator at 120 rpm at 37°C. After cultured for up to 80% spore formation, the cells were harvested by centrifuged at 4000 g to obtain pellet. The bacterial pellet was mixed with basal diet @ 5 × 10^8^ CFU/g. To achieve the well-distributed QSI-1 diet, the bacterial suspension was slowly added to feed power with gradual mixing in a laminar airflow bio-clean room. The mixed feed was oven-dried at 37°C for 3 h and stored at −20°C in sealed plastic ziploc™ bags until used. To ensure high QSI-1 level in the supplemented feed, fresh diets were prepared on weekly basis.

### Experimental design

Experimental treatments were carried out with randomly taken goldfish in six plastic aquaria (50 L capacity) at a density of 20 fish per aquarium. The experiments were run in triplicate. Fish were fed with basal diet or QSI-1 diet and equal amount were provided at 08:00 and 19:00 h for 15 days. The QSI-1 amount in the experimental feeds was adjusted approximately 4.0 × 10^8^ cells per g fish day. Control group were fed with the dry basal pellet without QSI-1. At the same time, fish health was also recorded by visual observation of behavior.

### Gut content sample processing

MS-222 (tricaine methane-sulfonate, Sigma, USA) at the concentration of 100 mg L^−1^ was used to anesthetize the fish. After dissection, the whole intestines of each fish were removed from the body under sterile conditions. Gut content samples were collected and immediately stored at −20°C. To isolate bacterial cells, the whole intestine from the fishes in the same group were put together and cut into small pieces and homogenized in ~10-fold dilution of sterile 1 × phosphate-buffered saline solution using autoclaved homogenizer, which made it possible to isolate most of the gut wall-attached microbes (Dawood et al., 2016).

### Intestinal bacterial counts

Fish gut content were sampled in each group after feeding for 14 d to determinate the total intestinal microbiota, *Bacillus* spp. lactic acid bacteria (LAB), *Escherichia coli* and *Aeromonas* spp. The fish were taken from the rearing tank onto a clean anatomical plate. The fish surface was sterilized with 70% ethanol before opening the abdominal cavity with sterile scissor. Intestinal tract was picked up, weighed and washed with PBS thoroughly three times. Samples were serially diluted with PBS and 100 μl of the different dilutions was spread onto triplicate TSA (Trypto-Soya agar) media to determine total bacterial populations. DeMan, Rogosa, and Sharpe agar media (MRS), *Bacillus* medium base and Eosin-Methylene blue media (EMB) to detect viable LAB species, *Bacillus* spp. and *E. coli* and *Aeromonas* spp. medium with ampicillin supplement (Ryan) were also used to detect *Aeromonas* species, all medium were purchased from Qingdao Hope Bio-Technology Co., Ltd, China. The agar plate inoculated with each dilution was incubated for 3–5 days at 28°C. Colony forming units (CFU ml^−1^) were determined for viable bacterial populations (Nikoskelainen et al., 2003).

### Detection of AHLs in fish gut by reporter strains

The AHL reporter strains *Chromobacterium violaceum* CV026 and *Agrobacterium tumefaciens* AT 136 (pZLR4) strains were used to detect the presence of short-chain and medium- to long-chain AHLs. For AT136 bioassay, 20 μl (50 μg/ml) 5-bromo-4-chloro-3-indolyl-b-D-galactopyranoside (X-gal) were spread on the LB plate before bioassay was performed. The AHL reporter strains was streaked on the LB agar plate. Then the gut content sample was placed approximately 0.5 cm apart from the streak. The plates were incubated at 28°C for 24 h. If the bacteria in the sample can produce AHLs, the AHLs can diffuse through the agar and activate the coloration of the biosensor strains.

### DNA extraction and pyrosequencing analysis of gut microbiota composition

The whole bacterial DNA was extracted from samples using the QIAamp DNA Stool mini kit (Qiagen, Germany) according to the manufacturer's protocol. In addition, samples were treated in FastPrep Lysing Matrix E tubes (MP Biomedicals, USA) three times for 60 s in a bead-beater (Mini-Beadbeater 8 Bio-Spec Products, USA) with one intervening minute on ice. The DNA concentration was determined by a Nano-Drop 1000 spectrophotometer (Thermo Scientific Inc., Wilmington, DE, USA). Universal primers (341F 5′-CCTAYGGGRBGCASCAG-3′ and 806R 5′-GGACTACNNGGGTATCTAAT-3′) targeting the V3-V4 region of 16S rRNA gene were chosen for the amplification of the PCR products and analyze the taxonomic composition of the bacterial community (Baker et al., 2003). The progress of the PCR amplification consisted of an initial denaturation step at 95°C for 4 min, followed by 25 cycles, where 1 cycle consisted of 94°C for 30 s (denaturation), 56°C for 30 s (annealing), 72°C for 60 s (extension), and a final extension of 72°C for 10 min. The PCR products were visualized on agarose gels (2% in TBE buffer) containing ethidium bromide. The 16S rRNA gene was analyzed to evaluate the bacterial composition by using Illumina Miseq (Majorbio Bio-Pharm Technology Co., Ltd. in Shanghai, China).

### Bioinformatic analysis of sequencing data

Pyrosequencing paired end reads were assigned, truncated and merged using Pandaseq software (V 2.7) to generate raw tags. High quality clean tags from the raw tags were obtained and compared with reference database to detect and remove chimeric sequences using Usearch (vsesion 7.1 http://drive5.com/uparse/) to obtain effective tags. QIIME platform (http://qiime.org/scripts/assign_taxonomy.html) was used to analyze the effective tags, and tags with ≥ 97% similarity were assigned to the same operational taxonomic unit (OTU). The phylogenetic affiliation of each 16S rRNA gene sequence was analyzed using the RDP Classifier (version 2.2 http://sourceforge.net/projects/rdp-classifier/) against the Silva (SSU115) 16S rRNA database, with a confidence threshold of 70% (Amato et al., 2013). Principal component analysis (PCA) and weighted Fast UniFrac principal coordinate analysis (PCoA) based on OTUs were performed to provide an overview of gut microbial dynamics in response to with or without probiotic treatments. Analysis of Community richness (Chao1 and Ace), community diversity (Shannon, Simpson), Sequencing depth (Good's coverage) and Observed species; and beta diversity on both weighted and unweighted unifrac distance metrics of OTUs were calculated with the MOTHUR program (http://www.mothur.org) (Schloss et al., 2009).

### Statistical analysis

Results were expressed as means and standard deviations. At least, three different measurements were accomplished for each mean. Statistical differences were determined using ANOVA, followed by Bonferroni's multiple comparison test. All statistical analyses were performed using Prism 6 (GraphPad Software, San Diego, CA, USA). A *t*-test was used to identify significant differences in relative abundance of bacterial taxa. *P* < 0.05 were considered significant.

## Results

### Culture-dependent analysis of bacterial population in the intestine of goldfish

**Figure 1:**
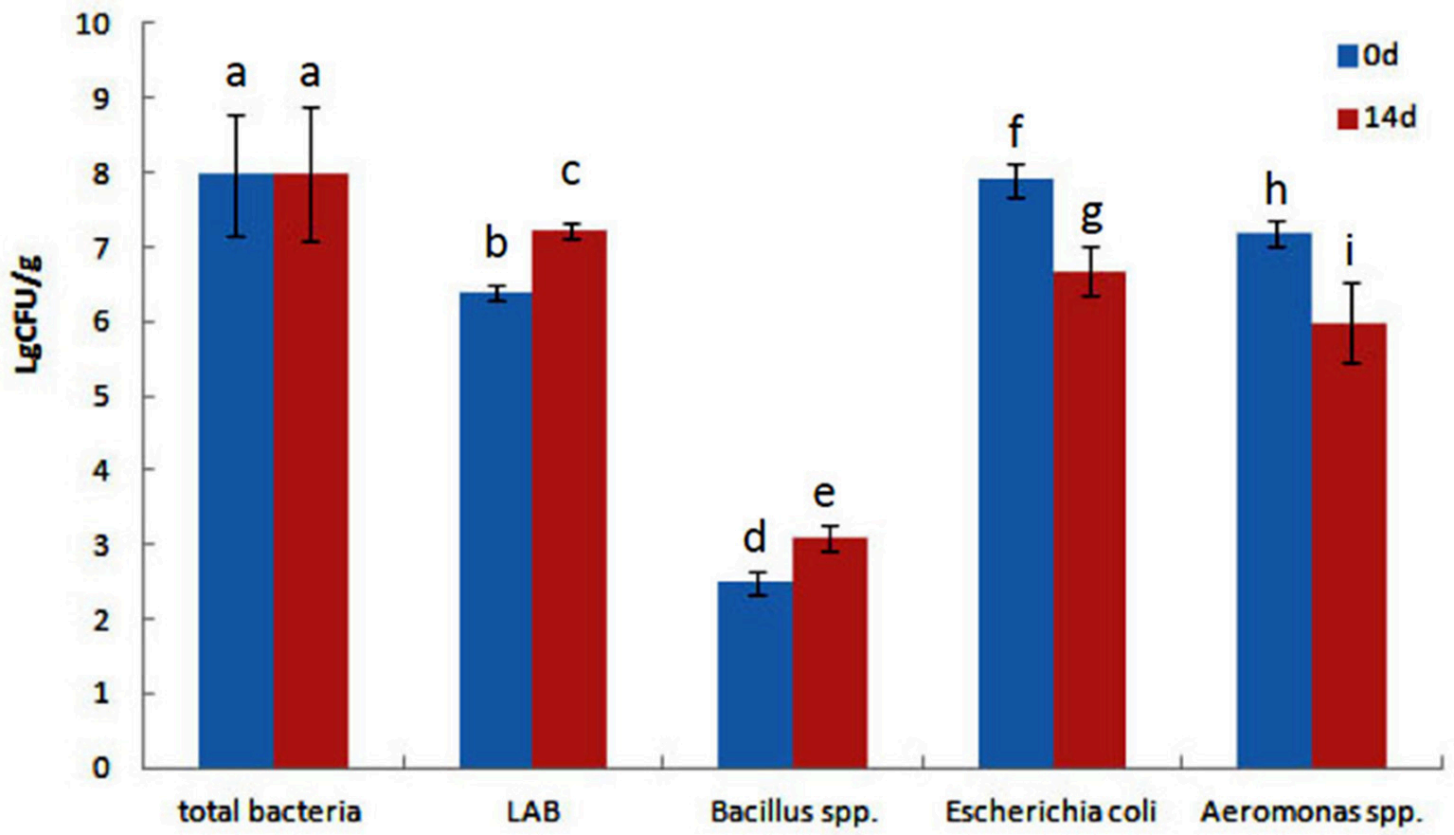
**Total culturable bacteria and lactic acid bacteria (LAB), ***Bacillus*** spp., ***Escherichia coli*** and ***Aeromonas*** spp. levels (log CFU g^**−1**^ intestine) of goldfish fed with diets with or without QSI-1**. Data represent means ± s.d. (*n* = 4 fish). Values with different letters are significantly different (*P* < 0.05). Values with the same letter are not significantly different (*P* > 0.05).

The effect of QSI-1 on the intestinal bacteria was determined using the traditional culture-based techniques. Total intestinal culturable microbiota, LAB species, *Bacillus* spp. *E. coli* and *Aeromonas* spp. from goldfish intestines were counted on TSA, MRS, *Bacillus* spp. EMB, and *Aeromonas* spp. selective media, respectively. Figure 1 shows the total viable cell counts, LAB, *Bacillus* spp., *E. coli* and *Aeromonas* spp. counts with the fish fed with or/without QSI-1 supplementations. No significant differences were observed in total bacteria levels between 0d and 14d with the levels around log 8 CFU g^−1^ (*P* > 0.05). LAB and *Bacillus* spp. levels were all significantly higher in the gut in the fish fed QSI-1supplemented diets (*P* < 0.05). But *E. coli* and *Aeromonas* spp. levels were all significantly lower in the gut after fed with QSI-1 for14 days (*P* < 0.05).

### Fish gut inhabitant AHLs producing *Aeromonas* spp

**Figure 2:**
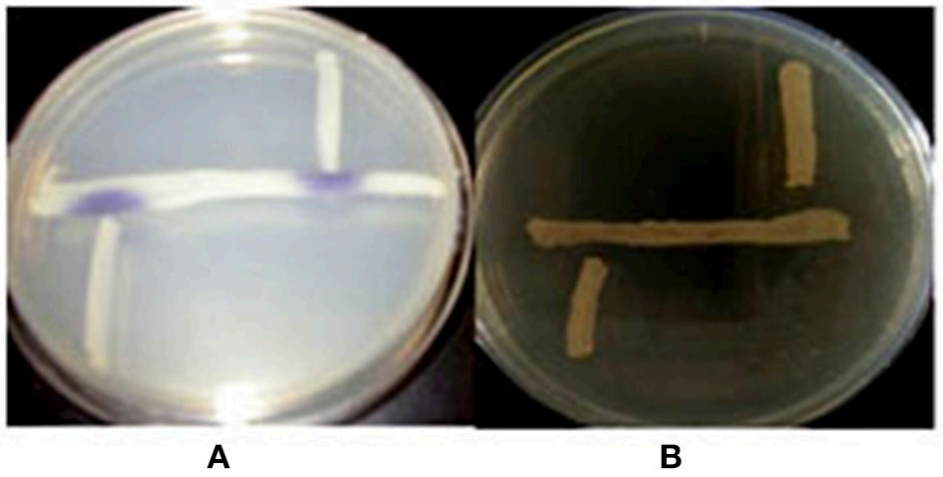
**The detection of AHLs production ***Aeromonas*** spp. isolates**. Cross-streaks of test strains (vertical) against biosensor strains (horizontal), **(A)** *C. violaceum* CV026, **(B)** *A. tumefaciens* AT 136 (pZLR4).

First, we determined whether QS signals are present in the environment of fish gut. We selected bacterial isolates from *Aeromonas* selective medium for its ability to produce AHLs by cross-streaking using biosensor strains. Violacein was induced in the *C. violaceum* CV026 AHL sensor and but did not stimulate the AHL sensor strain *A. tumefaciens* AT 136 (pZLR4) change to green, which responds to longer chain AHLs (Figure 2), it means the *Aeromonas* isolates can produce short-chain and medium AHLs but not long-chain AHLs.

**Figure 3:**
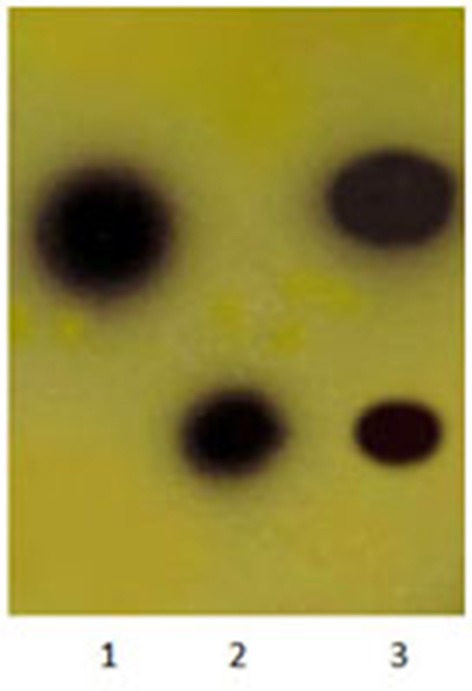
**Detection of AHLs by thin layer chromatography (TLC) with the ***C. violaceum*** CV026 strain used as a biosensor**. Lane 1, N-butanoyl-L-homoserine lactone (C4-HSL), lane 2, N-hexanoyl-L–homoserine lactone (C6-HSL), lane 3, *Aeromonas* spp. isolate extract.

Ethyl acetate extracts of AHLs from *Aeromonas* spp. isolates cultures retained the ability to induce reporter strains to produce pigment. AHLs extracts applied to TLC and visualized with the application of the *C. violaceum* CV026 sensor revealed the presence of two different AHLs in multiple extractions and chromatographs. On the basis of the mobility of the AHLs detected and comparison to synthetic standards, we tentatively identified these molecules as C4 HSL, and 3- C6 HSL (Figure 3).

### *Bacillus* sp. QSI-1 manipulate the AHLs levels in the fish gut

**Figure 4:**
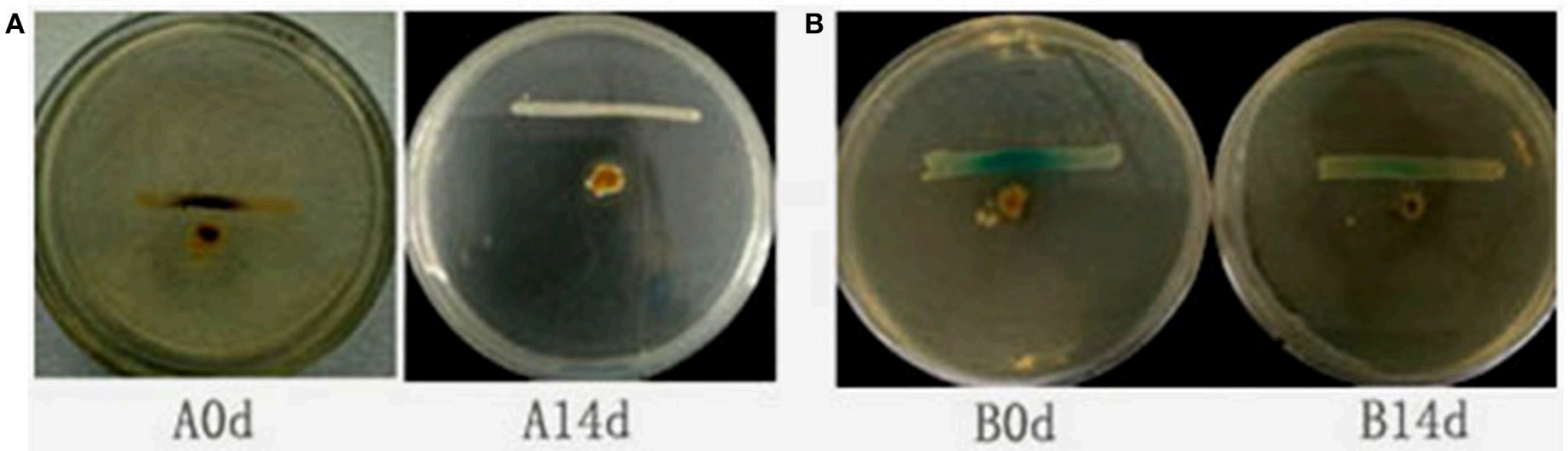
**The detection of AHLs in fish gut by cross-streaks of intestinal content (circle) against biosensor, (A)** *C. violaceum* CV026 and **(B)** *A. tumefaciens* AT 136 (pZLR4) (horizontal).

To determine whether QSI-1 can influence the production of AHL signal molecules in fish gut, QSI-1 was administered orally to fish for 14 days, and then the AHL signal molecules in the fish gut content were detected by reporter strains. Figure 4 showed that after 14 days orally administered of QSI-1, short-chain AHLs cannot be detected by CV026, and the medium- to long-chain AHLs were also decreased.

### *Bacillus* sp. QSI-1 alters the intestinal microbiota composition and reduce *A. hydrophila* level

**Figure 5:**
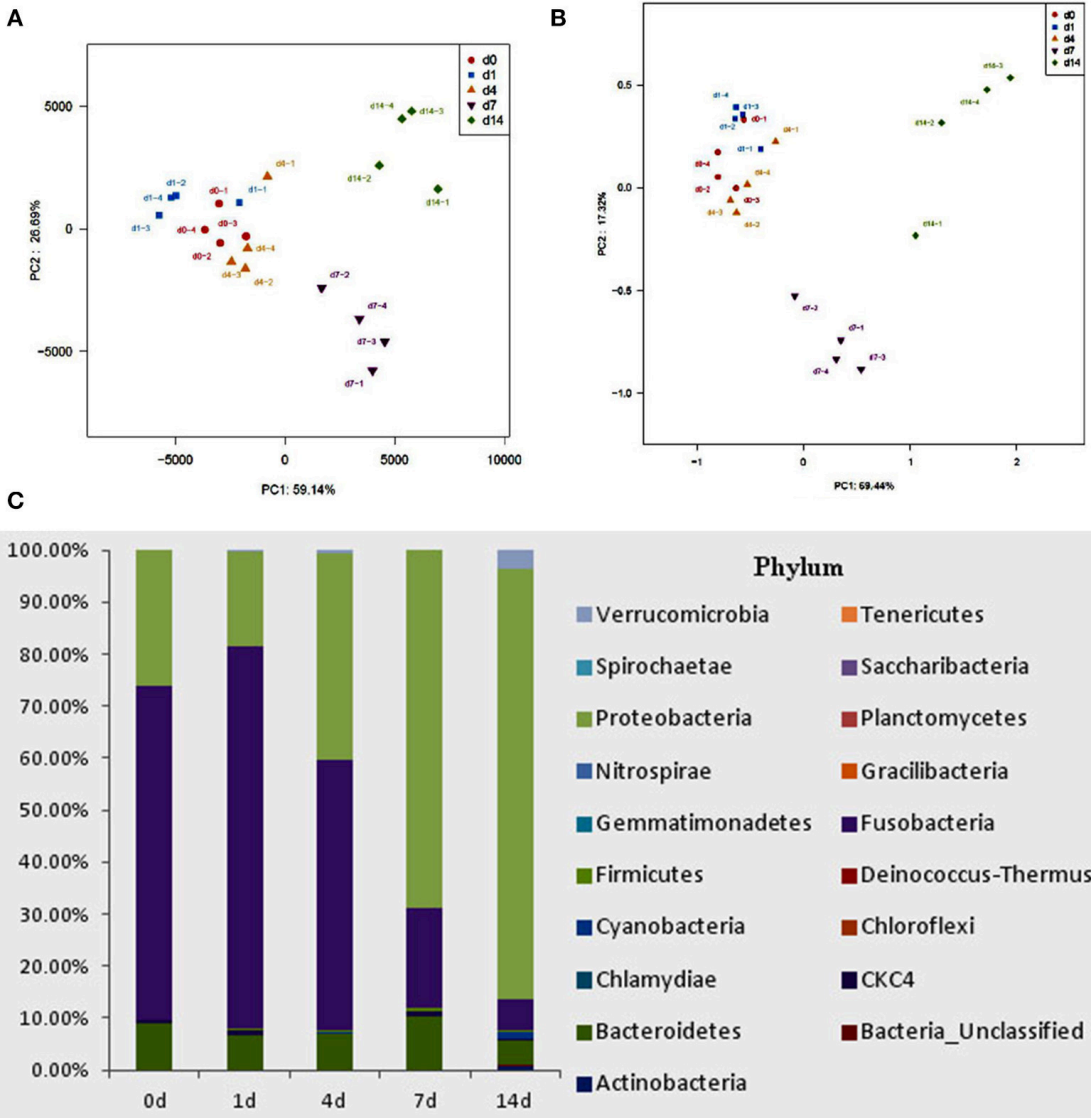
**Bacterial community analysis of goldfish gut content fed with or without QSI-1. (A,B)** principal component analysis (PCA) and principal coordinates analysis (PCoA) plots using Bray-Curtis metric and weighted Unicfrac distances, respectively. **(C)** Relative abundance of microbial genera (percentages) in the gut of fish fed with and without QSI-1 diets (*n* = 4).

High-throughput sequence analysis of bacterial 16S rRNA V3-V4 region was conducted on gut content samples. After filtering the low-quality reads, trimming the longer homopolymer runs, adapters, barcodes and primers, removing all cyanobacteria/chloroplast sequences and rarefying the datasets, the analysis revealed a highly diverse microbiota with a total of 760826 sequences (with an average length of 417.66 ± 6.5 bp), representing 316 OTUs, from 1702746 raw reads. All novel sequence data was deposited at NCBI's Sequence Read Archive under accession number SRP 093472. The relationships among samples was evaluated based on differences in phylogenetic diversity. Using the weighted-UniFrac metric, principal component analysis (PCA) and principal co-ordinates analysis (PCoA) plots were calculated from weighted UniFrac distances for the evaluation of the community composition, the results demonstrates a clear separation between each groups (Figures 5A,B), and suggesting that there is clear dissimilarity between the gut microbiota fed with or without QSI-1 supplemented diet. Figure 5C is the comparative presentation of the bacterial taxonomic analysis of intestinal microbiota composition between the groups with QSI-1 for 14 days and without QSI-1. At the phylum level, the bacterial communities in all treatments were dominated by three phyla: Bacteroidetes, Proteobacteria and Fusobacteria, but the higher relative abundance of Proteobacteria was observed after QSI-1 orally administrated. As shown in Table 1, differences were found in the diversity indices (Shannon and Simpson) and richness estimators (ACE and Chao) of the intestinal microbiota between the groups with QSI-1 for 14 days and without QSI-1.

**Table 1:** **Diversity estimation of the 16S rRNA gene libraries from microbiota in fish gut fed with normal diet and QSI-1 diets (mean ± s.d., ***n*** = 4)**.

**Group**	**Ace**	**Chao**	**Shannon**	**Simpson**	**Good's coverage**
0d	100.86 ± 24.59	79.23 ± 18.73	1.45 ± 0.10	0.34 ± 0.04	1.0 ± 0.0001
1d	173.50 ± 53.94	153.42 ± 47.81	1.33 ± 0.18	0.47 ± 0.11	1.0 ± 0.0003
*P* value	0.078	0.046	0.323	0.105	0.033
4d	185.20 ± 22.01	181.73 ± 22.22	1.74 ± 0.20	0.33 ± 0.04	1.0 ± 0.0001
*P* value	0.004	0.001	0.07	0.000009	0.005
7d	127.52 ± 21.92	115.73 ± 20.95	1.62 ± 0.19	0.33 ± 0.08	1.0 ± 0.0001
*P* value	0.211	0.066	0.221	0.786	0.108
14d	208.86 ± 41.28	205.69 ± 43.87	2.67 ± 0.56	0.19 ± 0.09	1.0 ± 0.0005
*P* value	0.008	0.004	0.01	0.033	0.058

**Figure 6:**
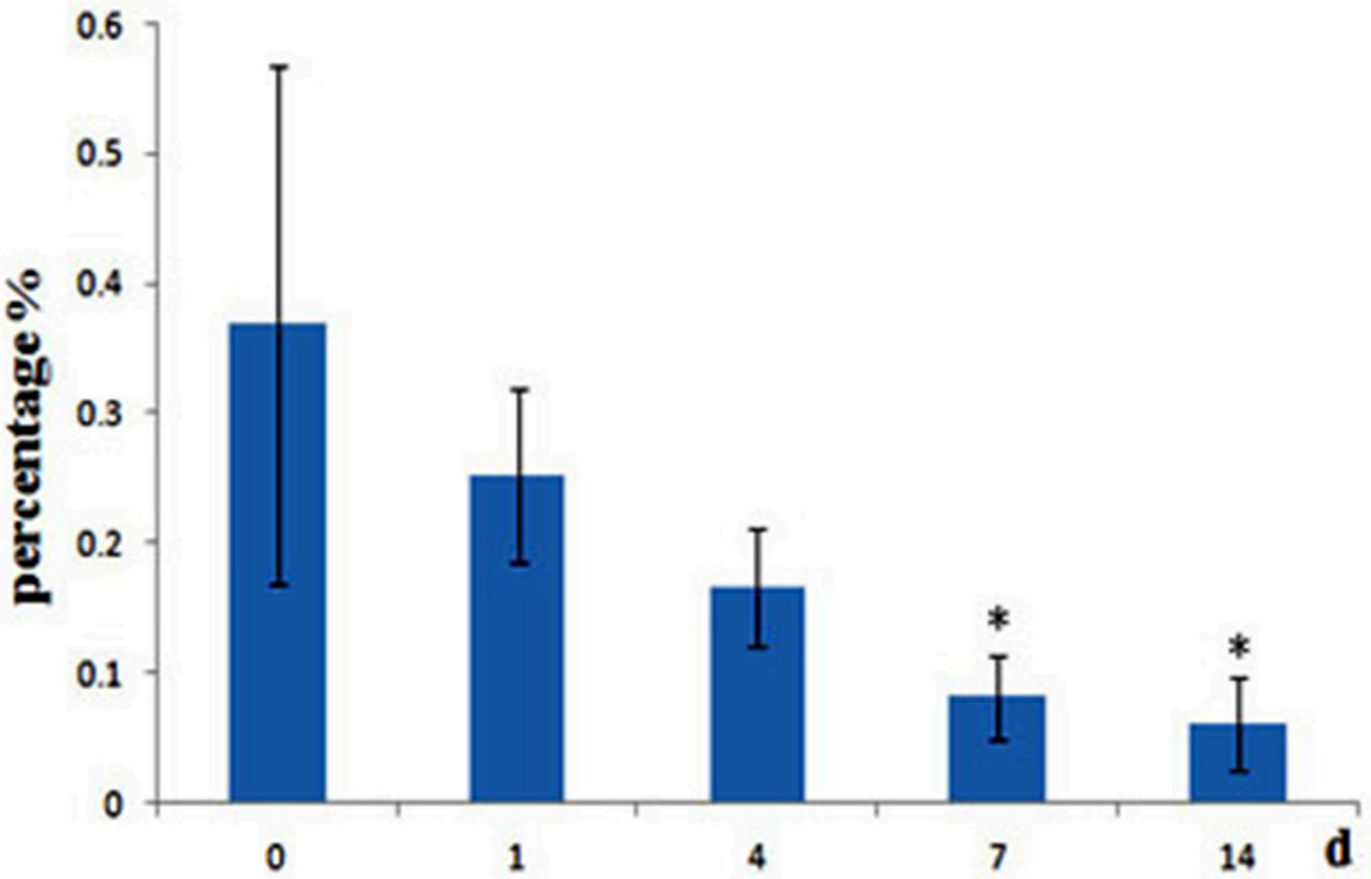
**Relative abundance of ***A. hydrophila*** in fish gut content after fed with QSI-1**. ^*^Means significantly different (*P* < 0.05) from the 0 day group.

*A. hydrophila* can produce two different short-chain AHLs, one is N-butanoyl-L-homoserine lactone (C4-HSL) and the other is N-hexanoyl-L-homoserine lactone (C6-HSL), so the level of *A. hydrophila* in fish gut was analyzed, we found that after administrated of QSI-1, the percentage of *A. hydrophila* in fish gut was decreased, and changed significantly at 7 and 14 days from 0.37 to 0.08 and 0.06% (Figure 6).

## Discussion

The fish intestine houses a dense and diverse microbial community, studies on fish gut microbiota have been reported by some researchers (Wu et al., 2013; Li et al., 2014; Kashinskaya et al., 2015; Gajardo et al., 2016), the gut microbiota is critical to health, and the microorganisms in the gut use chemical communication to coordinate and synchronize gene expression via the quorum-sensing regulatory pathway. Quorum sensing molecules, such as Autoinducer-2 (AI-2) and N-acyl-homoserine lactones (AHLs) are usually used by the gut microbiota (Borlee et al., 2008; Thompson et al., 2016). However, little is known about the acyl-HSLs being in the intestinal environment. In this study, using *A. tumefaciens* A136 and *C. violaceum* CV026 as report strains, we found that both short and long-chained AHLs are produced by gut microbiota in fish, and our findings also indicated that QS signals are active in the fish guts. These results are significant because AHLs are degraded by lactonolysis when the pH value above 7 and the open-ring AHLs is inactive (Yates et al., 2002; Funke et al., 2008). Borlee et al. (2008) study shown that bacteria from midgut of the cabbage white butterfly larval can produce AHLs and AHLs can be exchanged among bacteria in the alkaline environment and contribute to disease caused by *P. aeruginosa* PAO1. Some others also reviewed the different quorum sensing signal molecules within normal intestinal communities (Swearingen et al., 2013; Thompson et al., 2016). The pH value of gut alkaline environment is from pH 7.4 up to pH 8.2, while AHLs can still be detected. Perhaps it is due to bacteria residing in the fish gut alter the pH or by forming biofilms with other species in mixed species that reduce the pH.

The gut microbiota has been reported for other probiotics such as lactic acid bacteria, *Bacillus* spp. which modulate fecal microbiota (Cerezuela et al., 2013; Standen et al., 2015). Little is known about the influence of quorum quenching enzymes producing probiotics on the pathogens level and the structure of gut microbial community in fish. As far as we know, this is the first investigation to characterize the changes of gut microbiota of fish after administration of quorum quenching probiotics using classical cultural and high throughput methods. The gut microbial analysis demonstrated the dominant bacteria of Goldfish belonged to three phyla: Proteobacteria Bacteroidetes and Fusobacteria. These results are consistent with other studies on freshwater carp species, including grass carp and common carp (*Cyprinus carpio*) (van Kessel et al., 2011; Wu et al., 2012b; Ye et al., 2014; Li et al., 2015). Probiotics are live microorganisms which when are administered to animal confer a health benefit on the host, and usually been applied to enhance the immunity and disease resistance of fish. Probiotics can influence the composition and activity of the gut microbiota. Merrifield et al. (2010) suggested that probiotics provide several benefits to the host and modulate of the gut microbiota directly or indirectly. Standen et al. (2015) investigated the influence of a lyophilised probiotic mixture containing *Bacillus subtilis, Enterococcus faecium, Lactobacillus reuteri* and *Pediococcus acidilactici*, on the gastrointestinal microbiota of tilapia, their results shown that AquaStar® Growout can modulate both the intestinal microbiota and morphology of tilapia. Our results demonstrate that quorum quenching probiotics QSI-1 are capable of altering the gut microbiota structure and do so in specific manner by degrading AHLs. We also find that the concentration of fish pathogenic bacteria, *A. hydrophila* decreased. These results indicate that QSI-1 protect fish from *A. hydrophila* infection by quorum quenching pathway. Communication between different bacteria species can influence the interactions occurring in the environment. Thompson et al. (2015) show that the interspecies signal, autoinducer-2 (AI-2), can modulate the structure of the gut microbiota by using *E. coli* to manipulate AI-2 levels, and they also find that AI-2 could influence bacterial behaviors to restore the balance between the 2 major bacteria phyla, the Bacteroidetes and Firmicutes, following antibiotic treatment (Thompson et al., 2016).

In conclusion, our work indicates that oral administration of quorum quenching probiotics QSI-1 have positive effects on the gut microbiota structure and decreased *A. hydrophila* level by degrading AHLs. These results provide new insight into the mechanisms of probiotics, and suggest that quorum quenching probiotics can be used as an alternative strategy to combat bacterial infection in aquaculture instead of antibiotics.

## Author contributions

WC: Designed the experiments; SZ and AZ: Performed the experiments; HY: Analyzed the data; SZ and WC: Wrote the main manuscript text and AZ prepared figures. All authors reviewed the manuscript.

### Conflict of interest statement

The authors declare that the research was conducted in the absence of any commercial or financial relationships that could be construed as a potential conflict of interest.
